# The complete mitochondrial genome of drywood termite, *Incisitermes minor* (Isoptera: Kalotermitidae)

**DOI:** 10.1080/23802359.2017.1422397

**Published:** 2018-03-09

**Authors:** Yiyuan Liao, Haihong Chen, Shengli Lu, Yongjian Xie, Dayu Zhang

**Affiliations:** a The Key Laboratory for Quality Improvement of Agricultural Products of Zhejiang Province, College of Agricultural and Food Science, Zhejiang A&F University, Linan, China;; b Ningbo Housing Safety Management and Service Center, Ningbo, China;; c Yuyao Housing Safety Management and Service Center, Yuyao, China

**Keywords:** *Incisitermes minor*, Isoptera, mitochondrial genome

## Abstract

The complete mitochondrial genome of drywood termite, *Incisitermes minor*, is reported in this study. The circular mitogenome has a length of 15,970 bp and encodes 37 genes including 13 protein-coding genes (PCGs), 22 transfer RNA (tRNA), two ribosomal RNA (rRNA), and a non-coding control region (D-loop). The percentage of A and T (65.44%) within this mitogenome is much higher than that of G and C (34.56%). The phylogenetic tree revealed that mitogenomes of Kalotermitidae formed one clade. The tree also revealed that *I. minor* was closest to *Cryptotermes secundus*, and was a sister group to *Neotermes*. *I. minor* is a only species in which mitogenome has been completed so far among the *Incisiterm*es termite. The data provide resource for ecological and evolution analysis within termites especially Kalotermitidae.

*Incisitermes minor* (Isoptera, Kalotermitidae) is an important economic pest and invasive species (Cabrera and Scheffrahn 2017). *I. minor* also causes economic loses in China. *Incisitermes* termites include *I. minor* and other species such as *I. immigrans*, *I. incisus*, *I. marginipennis*, *I. synderi*, and *I. schwarzi* (Krishna and Weesner 1970). Although there are studies on the 16s ribosomal RNA (rRNA) mitochondrial gene of *I. minor* (Austin et al. 2012), there is no available information about its complete mitochondrial genome. The present study was the first report on the complete mitochondrial genome sequences of *I. minor* and added to the greatly increased insect mitogenomes (Cameron 2014).

Specimens were collected from Liyang, Ninghai County, Ningbo City, China and stored in the Insect Lab at Zhejiang A&F University, China (accession number NB0022-NH-1). The entire mitochondrial genome sequence of *I. minor* was 15,970 bp in length, including 13 protein-coding genes (PCGs), 22 transfer RNA (tRNA) genes, two rRNA genes, and one non-coding control region (D-loop). The genetic compositions and coding sequences are similar to other termites (Chen et al. 2016; Meng et al. 2016; Herve and Brune 2017). The L-strand codes 14 genes including four PCGs (*nad1, nad4, nad4L*, and *nad5*), eight tRNA genes (*trnQ, trnC, trnY, trnF, trnH, trnP, trnL1*, and *trnV*), and two rRNA genes (*rrnL and rrnS*). The other 23 genes were coded by the H-strand.

The overall sequences in the mitochondrial genome of *I. minor* were A + T biased. The A + T content of *I. minor* was 65.44%, higher than that of G + C (34.56%). The mitochondrial genome of *I. minor* included intergenic spacers and overlapping regions. The intergenic spacer sequences were spread on 17 regions ranging in size from 1 to 22 bp, and the overlapping sequences varied from 1 to 8 bp in eight regions.

Thirteen PCGs have a total length of 11,156 bp. There were 22 tRNA genes in the mitochondrial genome of *I. minor*. In addition to the tRNA-Ser which lacked the dihydrorubamide (DHU) arm, the other tRNAs all have the classical clover leaf structures. rRNA gene, *rrnL*, *rrnS*, is 830 bp and 1311 bp in length, respectively. *rrnS* is located between *trnV* and the control region, whereas *rrnL* is located between *trnL1* and *trnV*. The control region (D-loop) is located between *rrnS* and *trnI* gene, and is 1159 bp in length. The content of A + T in control region was 68.39%.

To understand the phylogenetic relationships among Kalotermitidae, a maximum likelihood phylogenetic tree was constructed using a dataset containing the nucleotide sequence of 13 PCGs of all mitochondrial genes (Bourguignon et al. 2015). *Microhodotermes viator* (Hodotermitidae) and *Zootermopsis nevadensis* (Archotermopsidae) were used as outgroups. On the tree, two major clades were formed, one clustering of *M. viator*, *Z. nevadensis*, and the other clustering of the remaining species which attach to the same family (Kalotermitidae) (Figure 1). This result was similar to the topology obtained from previous molecular studies (Bourguignon et al. 2015). The phylogenetic tree revealed that *I. minor* was closest to *Cryptotermes secundus*, and was a sister group to *Neotermes*.

**Figure 1.**
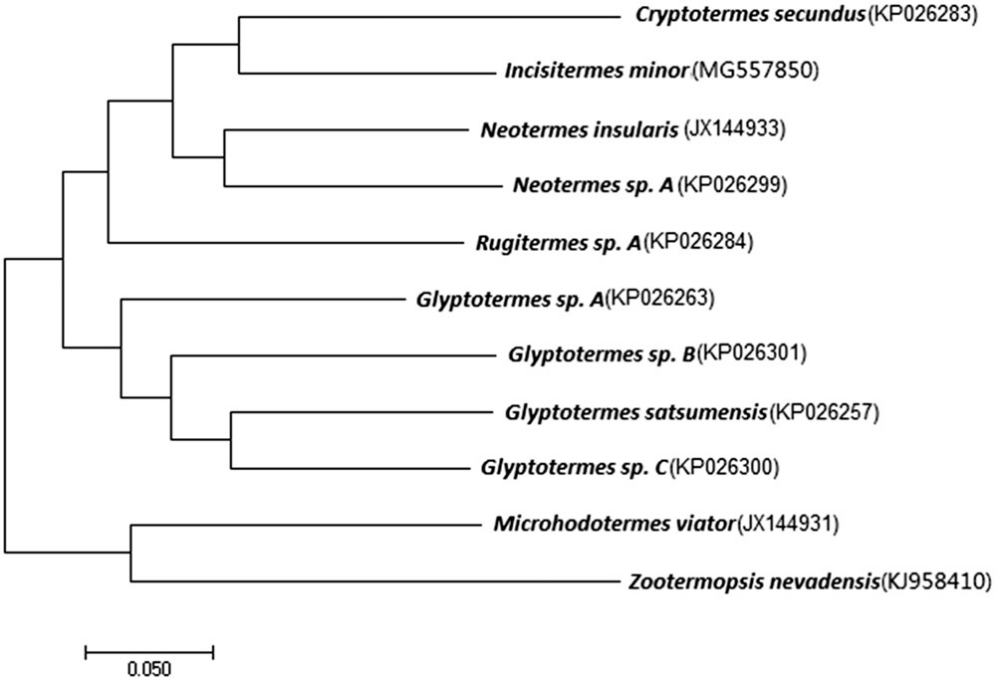
Maximum likelihood phylogenetic tree of selected termite mitogenomes including Kalotermitidae. The phylogenetic tree was built using 13 PCGs. Microhodotermes viator (Hodotermitidae) and Zootermopsis nevadensis (Archotermopsidae) were used as outgroups. Leaf names were presented as species names and GenBank accession numbers.

## Nucleotide sequence accession number

The complete genome sequence of *I. minor* has been assigned GenBank accession number (MG557850).
